# Role of 3 Tesla Magnetic Resonance Imaging in the Assessment of Infiltrative Cardiomyopathies

**DOI:** 10.7759/cureus.36719

**Published:** 2023-03-26

**Authors:** Tushar Kalekar, Arunima Gupta, Mudit Kumar

**Affiliations:** 1 Radiology, Dr. D. Y. (Dnyandeo Yashwantrao) Patil Medical College, Hospital and Research Centre, Pune, IND; 2 Radiodiagnosis, Dr. D. Y. (Dnyandeo Yashwantrao) Patil Medical College, Hospital and Research Centre, Pune, IND

**Keywords:** diastolic dysfunction, cardiac amyloidosis, cardiac sarcoidosis, infiltrative cardiomyopathy, cardiac mri

## Abstract

Background: The aim of the present study was to assess the role of 3 Tesla (3T) magnetic resonance imaging (MRI) in the assessment of infiltrative cardiomyopathy (ICM).

Methods: Cardiac MRI was performed on a 3T MRI machine for 15 patients who had clinical or echocardiographic signs of infiltrative cardiomyopathy. Each scan was assessed on a set of anatomical and functional parameters. The patterns of left ventricular (LV) late gadolinium enhancement (LGE) were also analyzed.

Results: Bi-atrial dilatation was noted in 14 patients, consistent with a restrictive phenotype. All 15 patients had diastolic dysfunction with reduced LV diastolic ventricular filling and prolonged peak filling times. Eleven patients had a decreased peak filling rate. Twelve patients had systolic dysfunction with reduced ejection fraction (EF). Ten patients had contractile dysfunction in the form of global LV hypokinesia. On delayed contrast imaging, four patients showed no abnormal LGE. Two patients showed diffuse subendocardial enhancement. Two patients showed patchy subendocardial enhancement. Six patients showed patchy mid-myocardial enhancement. One patient showed diffuse mid-myocardial enhancement. Three patients showed patchy subepicardial enhancement. Two patients showed patchy transmural enhancement. Three patients showed reversed myocardial nulling. All 15 patients received a provisional diagnosis of infiltrative cardiomyopathy on the basis of cardiac MRI findings. Sarcoidosis was given as a probable cause in four patients, amyloidosis in three patients, an infectious cause in two patients, and drug-induced cardiomyopathy in one patient. In five patients, no obvious cause could be identified.

Conclusion: Infiltrative cardiomyopathies, although relatively uncommon, pose significant challenges in diagnosis and treatment. Cardiac MRI has become the gold standard for non-invasive diagnosis of all infiltrative cardiomyopathies.

## Introduction

Cardiomyopathies are a significant contributor to cardiovascular mortality and morbidity worldwide [1]. They are primarily categorised into dilated cardiomyopathy (the most common), hypertrophic cardiomyopathy, restrictive cardiomyopathy (RCM), and arrhythmogenic right ventricular cardiomyopathy [2].

RCMs are a diverse group of diseases whose hallmark is diastolic dysfunction (due to restrictive filling) with relatively maintained systolic function [3]. They are classified according to their aetiology as either primary or secondary. Infiltrative cardiomyopathies (ICMs) are a subtype of secondary RCM characterised by the deposition of abnormal substances in the myocardium. They are relatively rare forms of cardiac disease and pose significant challenges in both diagnosis and treatment. These have varied aetiology and can be idiopathic, familial, or secondary to systemic disorders. Cardiac amyloidosis, sarcoidosis and hemochromatosis are the three most prevalent forms of ICM. Other forms of ICM include Fabry disease, Friedreich ataxia, and Danon disease [4].

Diagnosing ICM requires a high level of clinical suspicion; further investigations include transthoracic echocardiography (TTE), cardiac magnetic resonance imaging (MRI), and radionuclide imaging techniques such as positron emission tomography (PET) scans, and endomyocardial biopsy.

Cardiovascular magnetic resonance (CMR) imaging, with its superior spatial and tissue resolution, has become the major diagnostic modality in the evaluation of RCM and ICM. It is a non-invasive and non-ionizing imaging technique that has greatly revolutionised the diagnosis and follow-up of ICMs. It can definitively establish the diagnosis in suspected or clinical cases, assess the severity of the disease both qualitatively and quantitatively, check for complications and other systemic disease manifestations, provide prognostic information, and help in future follow-ups.

CMR can assess cardiac chamber size, morphology, and function and accurately quantify ventricular and atrial volumes. It can determine both systolic and diastolic function and calculate the ejection fraction (EF), end systolic volume (ESV) and end diastolic volume (EDV). Diastolic dysfunction, the defining feature of RCM, can be further assessed by ventricular filling on cine MRI and with parameters such as peak filling rate and peak filling time. Associated valvular dysfunction and wall motion abnormalities can also be observed. Features diagnostic of ICM on CMR include dilatation of both atria with relatively preserved ventricular size, thickening of the left ventricular (LV) wall, and diastolic dysfunction with relatively normal systolic function. However, advanced or severe cases of ICM show systolic dysfunction. Through its much larger fields of view, CMR can also demonstrate the involvement of the adjacent mediastinum and lungs in various ICMs.

The use of late gadolinium enhancement (LGE) has significantly improved the diagnostic capacity of CMR. Based on the LGE pattern, it is possible to differentiate various forms of infiltration and thus accurately establish the underlying aetiology.

The myocardial LGE pattern also helps to differentiate ICM from ischemic cardiomyopathy. LGE also provides prognostic information for different ICMs. CMR also allows for easy evaluation of the pericardium and can also differentiate between ICM and constrictive pericarditis. CMR can also help us conclusively rule out other differential diagnoses of dilated or hypertrophic cardiomyopathy in equivocal cases.

This study aims to assess the role of CMR imaging in the diagnosis of ICMs.

## Materials and methods

This descriptive observational study was conducted at Dr. D. Y. Patil Medical College, Hospital and Research Center, Pimpri, Pune, India, from August 2020 to July 2022, and 15 patients were included in the study. Clearance from the Institutional Ethics Subcommittee of Dr. D. Y. Patil Medical College, Hospital and Research Center was obtained beforehand (approval number: IESC/PGS/2020/171).

Inclusion and exclusion criteria

The following inclusion criteria were used: (i) Clinical suspicion or echocardiographic evidence of iICM, (ii) Suspicion of myocardial infiltration in known cases of systemic infiltrative diseases such as hemochromatosis, amyloidosis, or sarcoidosis, and (iii) Evaluation of the severity of cardiac involvement in known cases of ICM. Patients with MRI-incompatible metallic implants were not included in the study. Hemodynamically unstable, claustrophobic or uncooperative patients, and chronic kidney disease patients with a severely decreased estimated glomerular filtration rate (eGFR) were also excluded from the study. Pediatric patients with thalassemia major and suspected iron overload cardiomyopathy who underwent cardiovascular T2-star (T2*) imaging were also not included in the study.

Technique

A MAGNETOM Vida 3 Tesla (3T) MRI scanner (Siemens Healthineers, Erlangen, Germany) was used for the scans. A three-plane steady-state free precession (SSFP) localizer was taken initially to localise and plan the sequences.

Pre-Contrast Scanning

1. Sequences used for cardiac morphology: T2 true fast imaging with steady-state free precession (TRUFI) single shot, T2 half-Fourier acquisition single-shot turbo spin-echo (HASTE) dark blood, T1 turbo spin-echo (TSE) dark blood, T2 TSE dark blood, T2 short tau inversion recovery (STIR) dark blood long axis 

2. Sequences used for cardiac function: Cine sequences include two-chamber cine TRUFI long axis, Four-chamber cine TRUFI long axis, Cine TRUFI retro short axis, Cine left ventricular outflow tract (LVOT), and Cine right ventricular outflow tract (RVOT)

Post-Contrast Scanning

Post-contrast cardiac imaging was done using IV dimeglumine gadopentetate as the contrast agent (0.1 mmol/kg body weight). DYNAMIC TRUFI SR EPAT sequence was used for perfusion. For assessment of delayed enhancement, a short axis inversion scout sequence (TI scout) was taken 10 minutes post-gadolinium to identify the optimal TI value to null the signal from the normal myocardium. Based on the optimal TI value obtained, delayed TRUFI high-resolution phase-sensitive inversion recovery (PSIR) sequences were taken in two-chamber view, four-chamber view, and short axis view to obtain both magnitude and real images.

Retrospective electrocardiogram (ECG) gating was used for all image acquisition. Each scan was assessed on set anatomical and functional parameters. The patterns of left ventricular LGE were also analysed.

## Results

**Figure 1:**
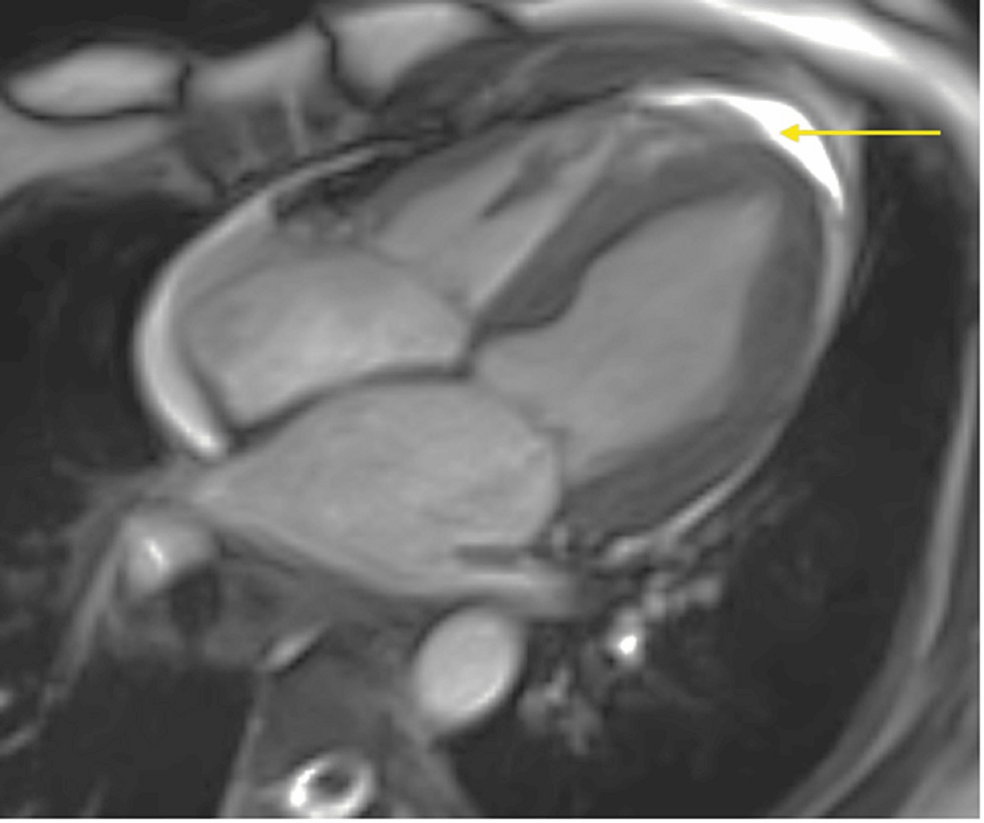
Four-chamber cine TRUFI long-axis view image of a 36-year-old female with cardiac sarcoidosis shows mild cardiomegaly with a dilated left ventricle and dilatation of both atria. Mild pericardial effusion is also noted (yellow arrow). TRUFI: true fast imaging with steady-state-free precession

A total of 15 patients were evaluated. The mean age was 49 years, ranging from 13 to 80 years. Seven patients were males (47%), and eight were females (53%). Fourteen patients had cardiac symptoms, the most common being dyspnea. One patient had no cardiac symptoms but was a known case of multiple myeloma. Seven patients had mild cardiomegaly. Seven patients had moderate cardiomegaly. One patient had gross cardiomegaly. Right and left atria were dilated in 14 patients, consistent with a restrictive phenotype (Figure 1). One patient had dilatation of the left atrium only. The left ventricle was dilated in eight patients. Six patients had a normal-sized LV cavity. One patient had a small LV cavity secondary to gross LV hypertrophy.

Eleven patients had normal LV wall thickness (≤ 12 mm). Four patients had LV hypertrophy, with a mean LV wall thickness of 20.5 ±9.34 mm (range, 13-37 mm). Twelve patients had systolic dysfunction with reduced EF (EF ≤ 55%), with the mean EF being 36.25±9.96% (range, 19-48%). Three patients had normal systolic function with preserved EF. All 15 patients had diastolic dysfunction, as ascertained by the visual evaluation of reduced LV diastolic ventricular filling on cine MRI images. All 15 patients showed prolonged peak filling times, with mean value being 498.26 milliseconds (ms), range 346-818 ms (normal: 135-212 ms). Eleven patients had a decreased peak filling rate (PFR < 2.4 EDV). Four patients had normal PFR (2.4-3.6 EDV).

**Figure 2:**
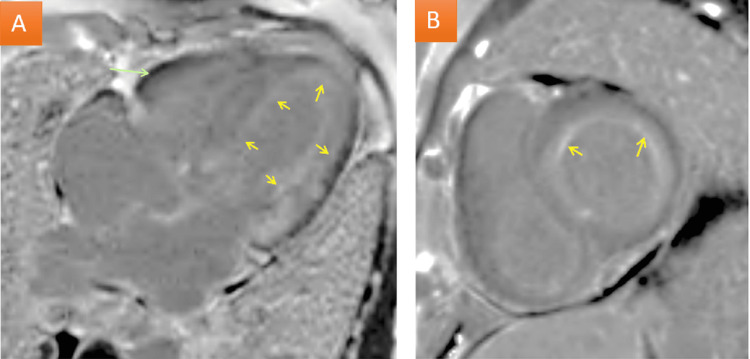
Delayed-enhancement (A) four-chamber long-axis and (B) short-axis images in a 64-year-old male with cardiac amyloidosis show diffuse subendocardial enhancement (yellow arrows) of left ventricle from base to apex involving all the cardiac segments. Associated mild pericardial effusion is also noted.

**Figure 3:**
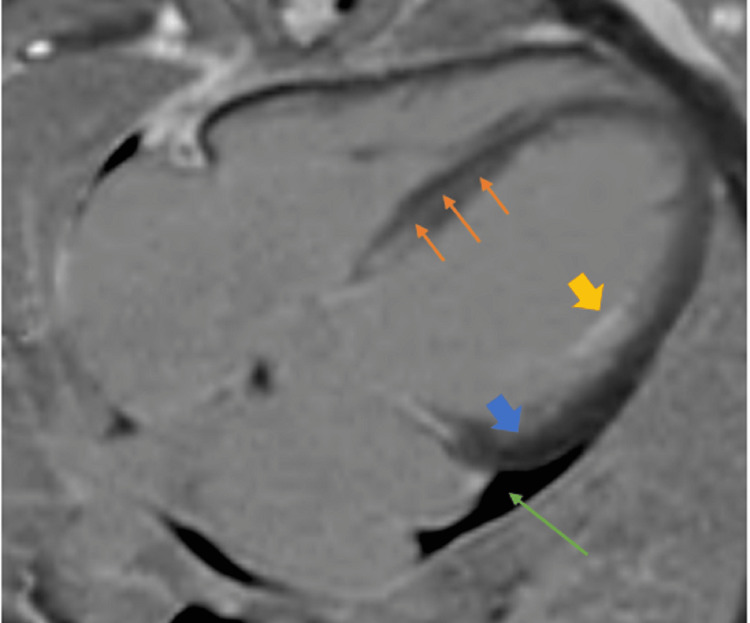
Delayed-enhancement four-chamber long-axis image in a 45-year-old male with drug-induced infiltrative cardiomyopathy shows mid-myocardial enhancement of the anterior septal and inferior septal segments of the mid and basal cavities (orange line arrows) and the inferior-lateral segment of the basal cavity (blue arrow). Patchy subendocardial enhancement of the mid-cavity along the lateral segment is also noted (yellow arrow). The presence of an associated mild pericardial effusion is also noted (green line arrow).

**Figure 4:**
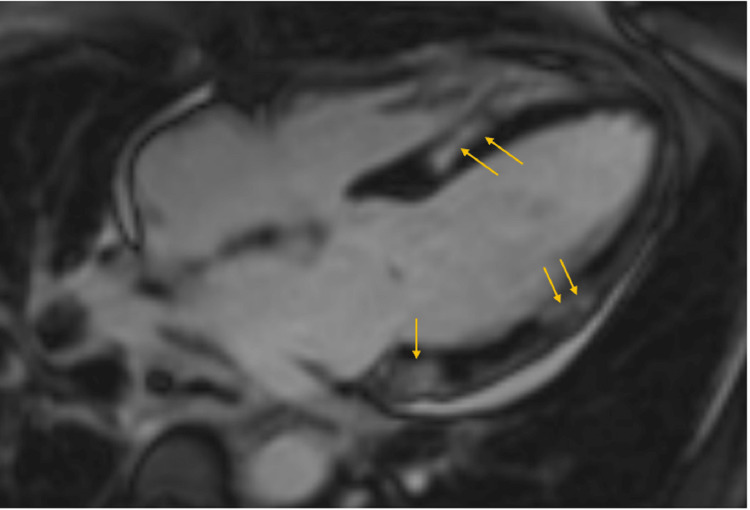
Delayed-enhancement four-chamber long-axis image showing patchy mid-myocardial and epimyocardial enhancement in the inferior, inferoseptal, and inferolateral walls of the basal and mid cavities in a female patient with cardiac sarcoidosis.

Valvular dysfunction in the form of mitral or tricuspid regurgitation was seen in 13 patients. Five patients had no wall motion abnormality. Ten patients had contractile dysfunction in the form of global LV hypokinesia. On evaluation of LV LGE patterns, four patients showed no abnormal LGE. Two patients showed diffuse subendocardial enhancement (Figure 2). Two patients showed patchy subendocardial enhancement (Figure 3). Six patients showed patchy mid-myocardial enhancement (Figure 3 and Figure 4). One patient showed diffuse mid-myocardial enhancement. Three patients showed patchy subepicardial enhancement. Two patients showed patchy transmural enhancement. 

**Figure 5:**
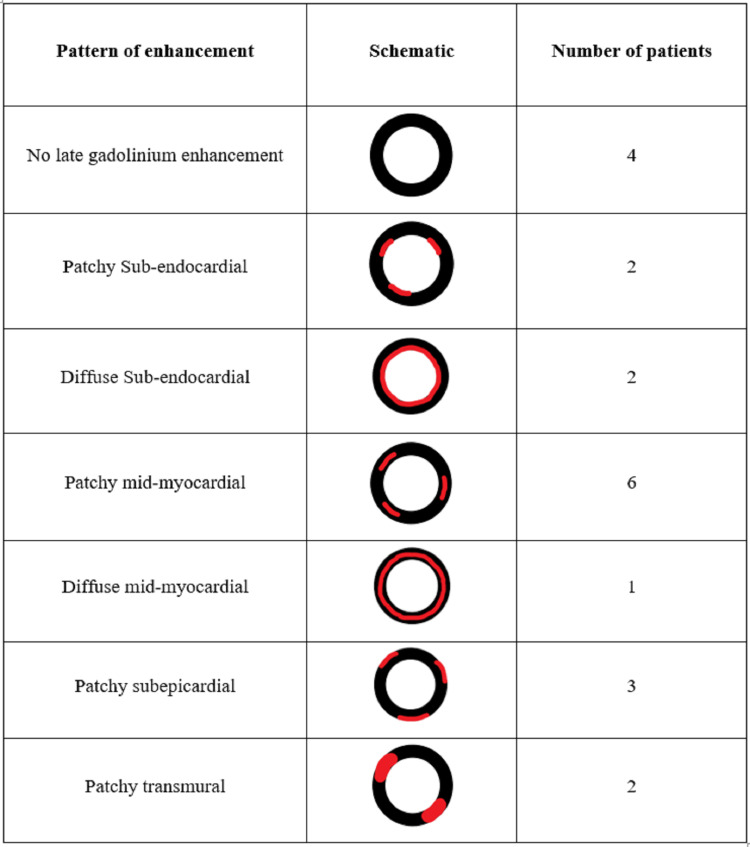
Distribution of patterns of left ventricular late gadolinium enhancement (LGE) among the study subjects

Figure 5 summarizes the distribution of patterns of LV LGE among the study subjects. In patients showing LV LGE, one patient showed LGE in three segments; the other 10 patients showed LGE in more than three segments. LGE in nine patients also involved the interventricular septum. In five patients, the right ventricle also showed LGE.

**Figure 6:**
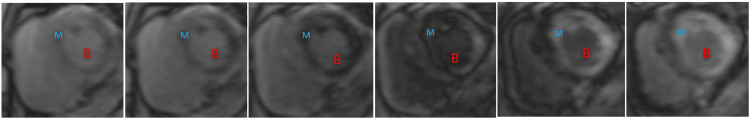
Sequential images from inversion scout sequence in a male patient with cardiac amyloidosis showing reversed nulling pattern with myocardium (M) nulling before blood (B).

Reversed myocardial nulling was seen in three patients (Figure 6), all of whom were given a provisional diagnosis of cardiac amyloidosis. 

**Figure 7:**
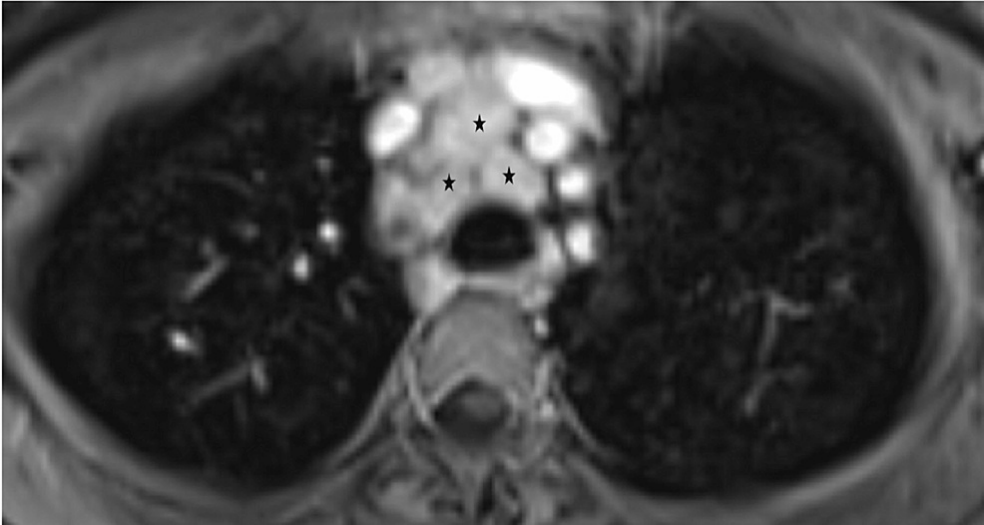
Mediastinal lymphadenopathy (black star) in a patient with cardiac sarcoidosis.

Pericardial effusion was seen in 10 patients (Figures 1, 2, 3). Pericardial thickening and enhancement were seen in one patient. In addition, no patient had any intracardiac thrombus or clot. Five patients showed bilateral pleural effusions. Three patients had mediastinal lymphadenopathy (Figure 7), one of whom also had abdominal lymphadenopathy. Two patients showed pulmonary consolidation. 

All 15 patients received a provisional diagnosis of ICM on cardiac MRI. Based on the pattern of enhancement, the presence or absence of reversed myocardial nulling, and other associated pulmonary and mediastinal findings, sarcoidosis was given as a probable cause in four patients, amyloidosis in three patients, an infectious cause in two patients, and drug-induced cardiomyopathy in one patient. In five patients, no obvious cause could be identified.

## Discussion

In recent years, CMR has become the major diagnostic modality in the evaluation of RCM and ICM. Cardiac MRI in ICM primarily shows dilatation of both the atria and diastolic dysfunction [3]. In our study, both right and left atria were dilated in 14 patients, consistent with a restrictive phenotype. One patient had dilatation of the left atrium only.

The LV wall can be thickened in ICM (LV wall thickness > 12 mm) because of the underlying infiltrative process, such as in amyloidosis, or it can be normal [3]. Ventricular cavity size may be relatively normal or the LV cavity may be dilated. Associated valvular dysfunction in the form of mitral or tricuspid regurgitation may be seen. In our study, 11 patients were found to have normal LV wall thickness (≤ 12 mm); four patients showed increased LV wall thickness.

Diastolic dysfunction, a chief criterion for ICM, can be assessed by a reduction in visual diastolic ventricular filling on cine MRI images, a prolonged peak filling time, and a decreased peak filling rate. Systolic function is relatively normal in cases with ICM. However, severe or advanced cases often show reduced LV systolic function. Global or regional wall motion abnormalities are also seen in many cases of ICM due to wall infiltration.

In our study, all 15 patients had diastolic dysfunction, as ascertained by the visual evaluation of reduced LV diastolic ventricular filling on cine MRI images. All 15 patients showed prolonged peak filling times, with mean value being 498.26 milliseconds (ms), range 346-818 ms (normal: 135-212 ms). Eleven patients had a decreased peak filling rate ( < 2.4 EDV). Four patients had normal peak filling rates (2.4-3.6 EDV). These suggest that peak filling time and peak filling rate can be used as useful CMR indicators for diastolic dysfunction. This is consistent with multiple studies that have found that a lower peak filling rate and a longer time to peak filling rate are useful indicators of diastolic dysfunction on CMR [5,6].

The absence of features such as pericardial thickening, septal flattening, or inversion on CMR helps in definitively differentiating ICM from constrictive pericarditis [3]. The use of LGE has significantly improved the diagnostic capacity of CMR. LGE in ICM is typically non-territorial, as opposed to that seen in ischemic cardiomyopathy. Based on the LGE pattern, it is also possible to differentiate various forms of infiltration. Diffuse, global, and subendocardial LGE is considered virtually pathognomonic of cardiac amyloidosis [3,7]. Cardiac sarcoidosis typically shows patchy mid-myocardial and sub-epicardial LGE [3,8,9]. Table 1 summarizes the characteristic CMR findings in common ICMs.

**Table 1:** Characteristic CMR findings in common infiltrative cardiomyopathies CMR: cardiac magnetic resonance; LGE: late gadolinium enhancement; LV: left ventricular T2* time: Observed transverse relaxation time

Condition	CMR
Cardiac Amyloidosis	Global subendocardial LGE; Reversed myocardial nulling on T1 Scout images
Cardiac Sarcoidosis	Patchy mid-myocardial or sub-epicardial LGE
Hemochromatosis/Iron Overload Cardiomyopathy	Shortened T2* time
Fabry Disease	LGE of the basal segments of the anterolateral and inferolateral walls
Danon Disease	Subendocardial LGE sparing the septum
Friedreich Ataxia	Symmetric hypertrophy of the interventricular septum, increased LV mass index

The diagnosis of cardiac amyloidosis is based on strong clinical suspicion and aided by the use of multimodality imaging, including a focused echocardiogram, technetium pyrophosphate scan, and above all, a dedicated CMR [10]. According to the findings of Maceira et al. [7] and Vogelsberg et al. [11], the most common pattern of LGE in cardiac amyloidosis is diffuse, global, and subendocardial, with variable transmural extension. In our study, among three patients with cardiac amyloidosis, two showed diffuse subendocardial LGE. One showed patchy subendocardial enhancement.

The presence of myocardial amyloidosis might be identified using the reversed nulling pattern on the TI scout sequence CMR, according to research by Pandey et al. [12]. They observed that in cardiac amyloidosis, CMR shows nulling of the myocardium before the blood pool, in contrast to the normal pattern, where the blood pool is nulled first and the myocardium later. In our study, reversed myocardial nulling was seen in three patients, all of whom were given the diagnosis of cardiac amyloidosis.

Additional findings described in cardiac amyloidosis include thickening and LGE of the atria [3], and thickening of the right ventricular (RV) free wall and atrial septum [13]. In our study, one patient with cardiac amyloidosis showed thickening of the RV wall. Atrial involvement was not seen in any patient.

In their study evaluating the role of CMR in cardiac sarcoidosis, Matoh et al. found LGE in five out of 12 patients [14]. In our study, among four patients with a diagnosis of sarcoidosis, one patient showed no LGE. The other three patients showed patchy intramural and subepicardial LGE involving multiple myocardial segments of the basal cavity, mid-cavity, and apical cavity, including the interventricular septum. This is consistent with the findings described by Vignaux et al. [8] and Ichinose et al. [9] that myocardial sarcoidosis shows diffuse or focal mid-myocardial or subepicardial LGE. A study conducted by Hulten et al. revealed that the presence of LGE is associated with an increased risk of future cardiovascular death and ventricular arrhythmia among cardiac sarcoidosis patients [15]. Patel et al., in a study of patients with biopsy-proven sarcoidosis, found that patients with myocardial damage (LGE) on CMR had a nine-fold higher rate of adverse events and an 11.5-fold higher rate of cardiac death than patients without damage [16].

Additionally, Vignaux et al. discovered that congestive cardiomyopathy with heart failure and widespread myocardial thickening with diffuse contraction anomalies may occasionally result from large infiltration with severe cardiac involvement [8]. In our study, three out of four patients diagnosed with cardiac sarcoidosis showed characteristic LGE involving multiple segments and wall motion abnormalities in the form of LV hypokinesia. All four of them showed systolic dysfunction with reduced EF.

Multiple associated extra-cardiac findings are often helpful in strengthening the diagnosis of cardiac sarcoidosis in patients with characteristic LGE patterns on CMR. Intrathoracic lymphadenopathy is the most common finding in sarcoidosis [17]. In our study, among the four patients with a diagnosis of sarcoidosis, three showed associated mediastinal lymphadenopathy. Pleural involvement is considered uncommon in sarcoidosis, with only 5% of patients showing pleural effusions on chest x-rays [18] and 8.2% of patients on CT [19]. In our study, a small volume pleural effusion was noted in one patient with cardiac sarcoidosis. Alveolar consolidation is found in 12-38% of sarcoidosis patients [20]. In our study, pulmonary consolidation was noted in one patient with cardiac sarcoidosis. Extrapulmonary involvement of sarcoidosis is reported in 30% of patients, with abdominal lymphadenopathy noted in 30% of patients [21]. In our study, one patient with cardiac sarcoidosis showed upper abdominal lymphadenopathy.

All 15 patients evaluated in our study received a provisional diagnosis of ICM on cardiac MRI. Among these, 12 patients had been identified as having a restrictive phenotype on two-dimensional (2D) echocardiography. This indicates that cardiac MRI shows 100% sensitivity and specificity in diagnosing ICMs and can further help in characterizing the aetiology. One patient had been given a diagnosis of constrictive pericarditis on 2D echocardiography. On cardiac MRI, however, he was diagnosed with ICM mostly secondary to infection (myopericarditis), in view of pericardial thickness < 4 mm and the absence of diastolic interventricular septal flattening. This is consistent with the findings of Farcone et al., who found early diastolic septal inversion or flattening at the onset of inspiration in all cases of constrictive pericarditis and in no case of RCM [22].

One patient had been diagnosed with hypertrophic obstructive cardiomyopathy on 2D echocardiography. Cardiac MRI, however, indicated findings suggestive of ICM, with dilatation of the left atrium and left ventricle, global hypokinesia, and extensive patchy mid-myocardial and transmural scarring showing LGE in multiple segments of the basal, mid, and apical cavities. There was no obvious asymmetrical septal or LV wall hypertrophy. Also, no obvious systolic anterior motion of the mitral valve was noted.

A patient found to have only LV hypertrophy with systolic dysfunction on 2D echocardiography was suggested a diagnosis of RCM based on biatrial dilatation, prolonged peak filling time (546 milliseconds), decreased peak filling rate (< 2.4 EDV), and a reduction in visual diastolic ventricular filling on cine MRI images.

Our study had some limitations that may affect the generalizability and validity of our findings. Firstly, we had a small sample size that did not include all types of ICMs. Also, in our institute, patients with iron overload cardiomyopathy (generally paediatric patients with thalassemia major undergoing repeated blood transfusions) only undergo cardiac T2* imaging and not a detailed CMR scan due to cost concerns. Hence, they could not be included in the study. Secondly, we could not obtain histologic confirmation in most patients. Thirdly, the high cost of conducting the study, the relative lack of awareness about the importance of cardiac MRI in ICM among referring clinicians, and the inability of a few patients to hold their breath for sufficient periods of time, all contributed to patient attrition in the study. Therefore, further studies with larger and more diverse samples are needed to confirm our findings.

## Conclusions

ICMs, although relatively uncommon, pose significant challenges in diagnosis and treatment. Early detection is important to institute timely intervention and avoid irreversible damage. Cardiac MRI has become the gold standard for non-invasive diagnosis of all ICMs. Not only does it offer the advantage of being non-ionizing and highly reproducible, but it is also highly accurate, sensitive, and specific. It can help establish a definitive diagnosis in suspected cases and also identify the underlying aetiology. It can also assess disease activity and severity and check for complications. It also provides prognostic information and allows for the evaluation of treatment response. 
